# Using Salivary Nitrite and Nitrate Levels as a Biomarker for Drug-Induced Gingival Overgrowth

**DOI:** 10.3389/fcimb.2015.00087

**Published:** 2015-12-01

**Authors:** Erkan Sukuroglu, Güliz N. Güncü, Kamer Kilinc, Feriha Caglayan

**Affiliations:** ^1^Department of Periodontology, Faculty of Dentistry, Karadeniz Technical UniversityTrabzon, Turkey; ^2^Department of Periodontology, Faculty of Dentistry, Hacettepe UniversityAnkara, Turkey; ^3^Department of Biochemistry, TOBB University of Economics and TechnologyAnkara, Turkey

**Keywords:** nitric oxide, nitrite, nitrate, saliva, drug induced gingival overgrowth, biomarker

## Abstract

**Aim:** Drug-induced gingival overgrowth has a multifactorial nature and the pathogenesis is still uncertain. It has been suggested that Nitric Oxide (NO) might play a role in the pathogenesis of drug-induced gingival overgrowth due to the contribution of NO to immune response and matrix degradation. NO levels in biological fluids have been used as a diagnostic biomarker in many diseases. The aim of this study is to determine whether NO levels in plasma, saliva, and gingival crevicular fluid (GCF) can serve as a potential biomarker for the evaluation of drug-induced gingival overgrowth risk.

**Materials and Methods:** A total of 104 patients, receiving cyclosporine A (*n* = 35), phenytoin (*n* = 25), nifedipine (*n* = 26), or diltiazem (*n* = 18) participated in the study. The amount of gingival overgrowth was evaluated with two indices and was given as percentage. Periodontal clinical parameters including plaque index (PI), gingival index (GI), gingival bleeding time index (GBTI), and probing depth (PD) were also assessed. Saliva, GCF, and plasma samples were obtained from each participants. Nitrite and nitrate levels in saliva, GCF, and plasma were analyzed by Griess reagent.

**Results:** Salivary nitrite and nitrate levels in responders were significantly higher than those in non-responders in only phenytoin group (*p* < 0.05). Nitrite and nitrate levels of gingival crevicular fluid and plasma did not significantly differ between responders and non-responders in all study groups (*p* > 0.05). Salivary nitrite levels exhibited a significant correlation with PD, GBTI, severity of gingival overgrowth (%GO), and GCF volume (*p* < 0.05). Additionally, a strong positive correlation was detected between saliva and plasma nitrate levels (*p* < 0.005). However, both nitrite and nitrate levels in GCF and plasma demonstrated no significant correlation with clinical parameters, GO severity, and GCF volume (*p* > 0.05).

**Conclusion:** Salivary nitrite and nitrate levels could be used as periodontal disease biomarkers in phenytoin induced gingival overgrowth, and that saliva seems to have a better diagnostic potential than GCF and plasma for the evaluation of drug-induced gingival overgrowth risk. However, when all drug groups were considered, saliva nitrite and nitrate levels could not be used as a biomarker for drug-induced gingival overgrowth.

## Introduction

Drug-induced gingival overgrowth is a well-known side effect of anticonvulsant phenytoin, immunosupressant cyclosporine A (CsA), and antihypertensive calcium channel blockers. Although the structural characteristics and pharmacologic effects of each of these drugs are different, all of them can cause gingival overgrowth that generally has a similar clinical and histological appearance. The overgrowth normally begins at the interdental papillae and is usually confined to the attached gingiva, but it may extend coronally and interfere with the occlusion, mastication and speech, and may also compromise proper plaque control (Marshall and Bartold, 1999). The prevalence rate of gingival overgrowth is about 50% for phenytoin (Güncü et al., 2006) and 25–30% for CsA (Boltchi et al., 1999). This prevalence rate in patients treated with calcium channel blockers varies widely from 10 to 50% (Ellis et al., 1999; Güncü et al., 2007).

Not all patients receiving these drugs develop gingival overgrowth and the severity of overgrowth varies between patients. Although, many clinical and laboratory studies have been performed to clarify the underlying pathogenic mechanisms responsible for drug-induced gingival overgrowth, the etiology remains still undisclosed. Many risk factors including poor plaque control, gender, age of the patient, drug dosage, pharmacokinetic variables, and genetic predisposition have been identified in a number of studies, but it is not possible to determine the patients at high risk for developing gingival overgrowth (Seymour et al., 2000).

The essential feature of drug-induced gingival overgrowth is an increase in the connective tissue matrix and it results from disturbance of the intricate balance between synthesis and degradation of the extracellular matrix of the gingival tissues. Some of the suggested factors responsible for this imbalance are cytokines, inflammatory mediators, and growth factors (Trackman and Kantarci, 2004; Chae et al., 2006; Kantarci et al., 2006).

Nitric oxide (NO) is a gaseous free radical generated from the conversion of L-arginine to L-citrulline by nitric oxide synthase (NOS) (Jenkins et al., 1995). Three distinct isoforms of NOS are known and identified as endothelial NOS (eNOS), neuronal NOS (nNOS), and inducible NOS (iNOS). eNOS and nNOS release small amounts of NO for a short period of time following receptor stimulation and they play an important role in regulation of physiological processes including platelet aggregation, vascular relaxation, and immune modulation. iNOS produces large amount of NO, and is expressed in response to bacterial endotoxins and cytokines, such as interleukin-1β and TNF-α (Forstermann et al., 1994; Lyons, 1995). Distinct from eNOS and nNOS, iNOS is considered to act in pathological conditions and plays a detrimental role in the regulation of inflammatory reactions. These NO-mediated detrimental effects probably occur in combination with the action of metalloproteinases and collagenases, produced by activated macrophages, polymorphonuclear cells, and fibroblasts (Kröncke et al., 1997; Kendall et al., 2001).

It has been shown that NO takes part in the etiopathogenesis of many diseases, including periodontal disease (Ohashi et al., 1999; Batista et al., 2002). Increased production of NO can cause destruction of periodontal tissues (Batista et al., 2002). Based on the role of NO on both immune response and matrix degradation in the periodontal environment, it can be speculated that NO may play an important role in the pathogenesis of drug-induced gingival overgrowth. However, only few animal and clinical studies have investigated the relationship between NO and drug-induced gingival overgrowth (Fu et al., 2000; Gau et al., 2005; Rezaie et al., 2005; Gürkan et al., 2009). Fu et al. (2000) reported a decrease in gingival dimensions of CsA-fed rats after NO substrate (L-arginine) treatment compared to controls. In another animal study, Gau et al. (2005) demonstrated that CsA-treated rats have increased plasma nitrite/nitrate levels compared to control rats. In a recent study, Gürkan et al. (2009) showed higher iNOS and eNOS immunostaining in connective tissues from patients in the gingivitis and CsA induced gingival overgrowth groups compared to healthy and subjects without gingival overgrowth. However, no intergroup differences were reported regarding nitrite/nitrate levels in gingival crevicular fluid (GCF). There is no clinical human study evaluating the relation of salivary nitrite and nitrate levels to drug-induced gingival overgrowth in the literature. There is only one study conducted on rats, in which the relation of nitric oxide to nifedipine-induced gingival overgrowth and salivary gland function was assessed. It was suggested that nitric oxide mediated positive signal-transduction mechanisms in salivary glands may play an important role in nifedipine-induced gingival overgrowth (Rezaie et al., 2005).

Due to its reactivity and short half-life, direct measurements of NO from tissues and biological fluids are hard to perform. Although NO metabolites have a very short life, nitrite and nitrate are the relatively stable end products of NO oxidation (Lappin et al., 2000; Ugar-Cankal and Ozmeric, 2006). The total levels of nitrite and nitrate in biological fluids are generally used for adequate monitoring of the NO synthesis (Moshage et al., 1995). Based on the findings that showed a marked increase in NO synthesis in overgrown gingiva, measuring nitrite and nitrate levels in saliva, GCF, and plasma might be useful to understand the etiopathogenesis of drug-induced gingival overgrowth. To the best of our knowledge, there is no study evaluating the association of nitrite and nitrate levels in saliva, plasma, and GCF with drug-induced gingival overgrowth. Drug-induced gingival overgrowth may constitute speech, mastication, hygienic, and aesthetic problems. Thus, early diagnosis of drug-induced gingival overgrowth is very important and it needs to have a diagnostic biological marker to determine the risk of developing drug-induced gingival overgrowth in patients receiving these drugs, before the occurrence of gingival overgrowth. Therefore, the present study was conducted to evaluate nitrite and nitrate levels in plasma, saliva, and GCF from responders and non-responders treated with either CsA, phenytoin, nifedipine, or diltiazem, and to determine whether NO levels in plasma, saliva, and GCF can serve as a potential biomarker for the evaluation of drug-induced gingival overgrowth risk.

## Materials and methods

### Study population and design

This research study was conducted at the Department of Periodontology, Hacettepe University between February 2010 and September 2013. A total of 104 Turkish patients (50 male, 54 female) were included in the study. The study groups consisted of 35 renal transplant patients receiving CsA, 25 patients receiving phenytoin, 26 patients receiving nifedipine, and 18 patients receiving diltiazem therapy. Patients participated in this study were obtained from outpatients attending the Department of Nephrology, Neurology, and Cardiology, Faculty of Medicine, Hacettepe University. Only patients receiving one of these drugs (including CsA, phenytoin, nifedipine, and diltiazem) in regular doses for at least 6 months were included in the study. The presence of all maxillary and mandibular anterior teeth was also required. Pregnant women, subjects with systemic diseases such as diabetes mellitus or any other form of systemic inflammatory involvement, patients who have mouth breath and restorative crowns at anterior regions, and used any antibiotics or received periodontal treatment within the last 6 months and who also received one more drug leading to gingival overgrowth were excluded from the study. Due to the fact that NO levels could be affected by smoking, smokers were not included in the study. The protocol of the study was approved by the ethics committee of the Hacettepe University. (#FON 07/15-1) Written informed consent in accordance with the Declaration of Helsinki was obtained from each patient prior to enrollment in the study.

### Clinical parameters

Periodontal examinations were carried out on the upper and lower six anterior teeth by one calibrated periodontal specialist (ES) during the study period. Periodontal clinical parameters including plaque index (PI; Löe, 1967), gingival index (GI; Löe, 1967), probing depth (PD), and gingival bleeding time index (GBTI; Nowicki et al., 1981) were recorded. Demographic variables including age and gender were also recorded.

In order to assess the overgrowth of gingival tissues accurately, alginate impressions were obtained from each patient and gypsum individual models were created. Gingival overgrowth was assessed on these individual models by performing measurements in vertical and horizontal dimensions. Vertical measurements were performed according to the Miller and Damm index (1992). Briefly, the height of the gingival tissue was measured from the cemento-enamel junction (CEJ) to the free gingival margin. The following grades were scored in six points around each tooth on maxillary and mandibular anterior region: 0 = normal gingiva; 1 = minimal enlargement (≤2 mm in size); 2 = moderate enlargement (2–4 mm in size); and 3 = severe enlargement (nodular growth >4 mm). Horizontal gingival overgrowth was measured in the buccal-lingual direction of all interdental papillae of maxillary and mandibular anterior teeth according to Miranda et al. (2001). The increase in the size of buccal and lingual papillae was measured from the enamel surface to the outer papillary surface at the interdental contact point. Horizontal measurements were assigned a score of between 0 and 2, as follows: 0 = normal thickness (1 < mm); 1 = thickness between 1 and 2 mm; and 2 = thickness more than 2 mm.

The vertical and horizontal scores were calculated to determine an overgrowth score for each gingival papillae. The maximum overgrowth score obtained for each interdental unit was five. Twenty interdental units were identified, giving a maximum overgrowth score of 100 for a patient. Therefore, this index could be expressed as a percentage (Thomason et al., 2005). A score of 30% was determined as the critical value for gingival overgrowth index. Patients were classified into two subgroups (Seymour and Smith, 1991) as followed: patients with a gingival overgrowth index of < 30% were identified as non-responders (GO–) and patients with a value of ≥30% were defined as responders (GO+). The severity of GO was also determined with this percentage.

### GCF, saliva, and plasma sampling

The participants were told not to eat any high nitrate foods (e.g., spinach, beets, and other green leafy vegetables) at least 12 h before sampling due to the fact that it may affect the study results. GCF samples were obtained from maxillary anterior teeth. To avoid blood contamination and possible stimulation of GCF flow during clinical measurements, samples were collected before any other clinical recordings except the PI. GCF samples were obtained by the technique described by Rüdin et al. (1970). Prior to sampling, the respective region was dried by isolation with cotton rolls and a 5-s gentle air stream with a 90° angle to the tooth axis, and supragingival plaque was eliminated with a curette. Standardized strips were inserted 1 mm into the sulcus and left for 30 s. Papers with visible blood contaminations were discarded and sampling was replicated from another site of the natural tooth that was not sampled. To eliminate the risk of evaporation, paper strips with GCF were immediately transported to a previously calibrated electronic GCF measuring device for volume determination. The GCF was measured electronically in Periotron units, which were converted to micro liters (μl) by MCCONVRT software (Software version 2.52, Oraflow Inc., Amityville, NY, USA). GCF samples were then placed in sterile Eppendorf tubes and stored at −30°C until the day of laboratory analysis. A total of 10 paper strips with GCF were obtained with this standardize technique from maxillary anterior six teeth of each patient for detecting GCF nitrite and nitrate levels.

After GCF collection, stimulated parotid saliva samples were obtained from each patient using Lashley cup (Lashley, 1916) during stimulation with 1% citric acid. Citric acid was applied to the dorsum of the tongue, held there by the subject for 10 s and 1 ml of saliva was collected from each patient as described in detail before (Francis et al., 2001). Saliva samples were then placed in sterile Eppendorf tubes and stored at −80°C until the laboratory analysis.

After saliva collection, in order to determine the plasma nitrite and nitrate levels, venous blood samples were collected into lithium-heparin tubes. Blood samples were placed within 3 min into a refrigerated (4°C) centrifuge and centrifuged at 4000 rpm for 10 min according to standard procedures (Uzar et al., 2011). After getting plasma, samples were frozen at −80°C for later analysis of plasma nitrite and nitrate concentrations.

### Determination of nitrite and nitrate levels in GCF, saliva, and plasma

For the determination of nitrite and nitrate levels in GCF samples, 300 ml of phosphate buffer (50 mM, pH: 7.4) was added onto each sample and the samples were mixed vigorously for the extraction of nitrite and nitrate into buffer. Nitrite and nitrate content of the samples extracts was determined using Griess reagent (Grisham et al., 1996). The sum of nitrate and nitrite was measured by the same assay after reduction of nitrate to nitrite with nitrate reductase from *Aspergillus niger* (Roche Diagnostics, Mannheim, Germany). Nitrite and nitrate levels in both saliva and plasma were determined by the same method without any pretreatment. The nitrate concentrations were calculated by subtracting the nitrite level from the total nitrite level (nitrite + nitrate). This assay is in the routine research analyze protocol of the laboratories of Hacettepe University Faculty of Medicine Department of Biochemistry for more than a decade and its sensitivity and specificity is being tested routinely by the university staff, together with other routine tests. However, commercial standards were implemented to confirm its sensitivity and specificity in the present study.

## Statistical analysis

Statistical analysis was performed with (SPSS Inc.,Chicago, IL; Serial number: 2Z7ZHN12FA70P1JXWYNCMWY5). The normality of data was tested with Kolmogorov Smirnov test. Mann Whitney U test was used to compare each clinical or laboratory findings for individual drug group between responders and non-responders. Spearman correlation test was obtained to determine the interactions between laboratory and clinical parameters for all participants. The differences between the age groups were tested with Independent sample *t*-test, and the distribution differences of gender was analyzed with chi-square test interms of between responders and non-responders. The confidence interval was set as 95%.

All patients participated in this study (*n* = 104) revealed a power of 87.74% with a normal approximation method. A difference in salivary nitrate levels between responders and non-responders in phenytoin group can be detected at an alpha level of 0.05, with a statistical power of 87.74%.

## Results

A total of 104 patients consisting of CsA group (*n* = 35), phenytoin group (*n* = 25), nifedipine group (*n* = 26), and diltiazem group (*n* = 18) were included in the study. The demographic variables of groups including mean age and gender ratio are presented in Table 1.

**Table 1:** **Demographic variables of study population**.

	**Phenytoin (*n* = 25)**		**CsA (*n* = 35)**		**Nifedipine (*n* = 26)**		**Diltiazem (*n* = 18)**	
	**GO+ (*n* = 11, 44%)**	**GO− (*n* = 14, 56%)**	**GO+ (*n* = 13, 37.1%)**	**GO− (*n* = 22, 62.9%)**	**GO+ (*n* = 11, 42.3%)**	**GO− (*n* = 15, 57.7%)**	**GO+ (*n* = 2, 11.2%)**	**GO− (*n* = 16, 88.8%)**
Age	40.1 ± 12.2	47.7 ± 16.2	35.3 ± 9.6	41.0 ± 9.7	51.1 ± 8.9	50.3 ± 9.1	53.5 ± 4.9	57.5 ± 9.6
**Gender**								
Male	6	8	5	13	7	3	1	7
Female	5	6	8	9	4	12	1	9

Patients in each drug group were divided into two subgroups as responders (overgrowth index ≥30%) and non-responders (overgrowth index < 30%) according to gingival overgrowth index. Out of 35 patients in CsA group, 13 (37.1%) were identified as responders, those values in phenytoin, nifedipine, and diltiazem groups were 11 (44%), 11 (42.3%), and 2 (11.1%), respectively. The gingival overgrowth index was significantly higher in responders in all drug groups, as expected (Table 2). There were statistically no significant differences between responders and non-responders in mean age and gender ratio in all drug groups except for nifedipine. While the mean age was similar between subgroups in nifedipine group, there was a significant difference in gender ratio (*p* < 0.05).

**Table 2:** **Clinical periodontal parameters in all groups**.

**Clinical Periodontal Parameters**	**Phenytoin (*n* = 25)**		**CsA (*n* = 35)**		**Nifedipine (*n* = 26)**		**Diltiazem (*n* = 18)**	
	**GO+**	**GO−**	**GO+**	**GO−**	**GO+**	**GO−**	**GO+**	**GO−**
PD	2.26±0.57^*^	1.45±0.51	2.52±0.49^*^	1.45±0.32	2.28±1.02^*^	1.39±0.38	2.46±0.83	1.49±0.33
PI	1.05±0.58^*^	0.41±0.36	1.52±0.54^*^	0.62±0.48	0.90±0.63	0.56±0.46	1.22±0.74	0.59±0.44
GI	1.62±0.62^*^	0.88±0.52	2.04±0.38^*^	0.83±0.61	1.64±0.41^*^	0.79±0.57	1.82±0.25^*^	0.87±0.36
GBTI	2.24±0.91^*^	1.28±0.75	2.80±0.25^*^	1.17±0.98	2.29±0.55^*^	1.08±1.07	2.75±0.35^*^	1.10±0.73
%GO	53.0±11.93^*^	17.42±7.41	53.84±17.19^*^	18.36±5.99	44.81±12.2^*^	20.13±6.05	33.00±2.82^*^	18.81±5.77
GCF V(μl)	0.94±0.56	0.78±0.69	1.70±0.99^*^	0.74±0.44	1.24±0.71	1.00±0.69	1.61±0.26^*^	0.92±0.39

PD, probing depth; PI, plaque index; GI, gingival index; GBTI, gingival bleeding time index; %GO, percentage of gingival overgrowth; GCF V, Volume of gingival crevicular fluid.

* *p < 0.05*.

### Clinical periodontal parameters

All clinical periodontal parameters including PI, GI, PD, and GBTI were statistically higher in responders compared to non-responders in all drug groups (*p* < 0.05), except for diltiazem and nifedipine group (Table 3). In diltiazem group, these clinical parameters were higher in responders but only the differences in GBTI and GI reached statistically significance (*p* < 0.05). In nifedipine group, all clinical periodontal parameters except PI were statistically higher in responders (*p* < 0.05).

**Table 3:** **Nitrite and nitrate levels in saliva, plasma, and in GCF**.

	**Phenytoin (*n* = 25)**		**CsA (*n* = 35)**		**Nifedipine (*n* = 26)**		**Diltiazem (*n* = 18)**	
	**GO+**	**GO−**	**GO+**	**GO−**	**GO+**	**GO−**	**GO+**	**GO−**
Saliva nitrite level (μM)	7.08±6.82^*^	2.90±2.36	7.44±6.16	4.75±3.42	4.87±4.13	6.18±5.84	3.60±1.81	4.96±4.07
Saliva nitrate level (μM)	474±37.17^*^	421.30±47.23	456.72±54.05	478.69±52.85	475.65±55.96	430.63±65.02	372.23±79.26	424.09±47.78
Plasma nitrite level (μM)	4.33±4.69	3.13±1.76	4.30±3.15	2.60±2.17	3.74±2.82	3.00±2.54	6.16±4.89	4.84±3.76
Plasma nitrate level (μM)	27.89±9.49	26.77±10.58	32.24±18.60	42.89±20.43	32.45±7.18	26.21±7.90	25.65±12.11	27.22±12.97
GCF nitrite level (nmol)	6.39±3.64	5.12±4.72	7.51±4.38	9.17±5.07	7.27±5.39	6.21±4.94	7.94±3.11	4.28±2.91
GCF nitrate level (nmol)	226.22±47.57	253.96±75.61	215.91±64.58	221.01±49.20	289.09±47.78	288.83±64.40	231.93±34.02	253.70±53.65

* *p < 0.05*.

### Nitrite and nitrate levels in saliva, GCF, and plasma

Nitrite and nitrate levels of saliva, GCF, and plasma for the subgroups of each study group are shown on Table 3. Salivary nitrite and nitrate levels (μM) did not differ significantly between responders and non-responders in all study groups, except for phenytoin group. In phenytoin group, salivary nitrite and nitrate levels in responders were significantly higher than those in non-responders (*p* < 0.05). Nitrite and nitrate levels of GCF (nmol) and plasma (μM) did not significantly differ between responders and non-responders in all study groups (*p* > 0.05).

Correlations between saliva, GCF, and plasma nitrite/nitrate levels, periodontal clinical parameters, severity of GO, and GCF volume were presented in Table 4. Salivary nitrite level exhibited a significant correlation with PD, GBTI, severity of GO (%GO), and GCF volume (*p* < 0.05). There was no significant correlation between salivary nitrate level and periodontal clinical parameters, except PD. A strong positive correlation was detected between salivary and plasma nitrate levels (*p* < 0.005). There was a negative significant correlation between nitrite and nitrate levels in GCF (*p* < 0.05). Both nitrite and nitrate levels in GCF and plasma demonstrated no significant correlation with clinical parameters, GO severity, and GCF volume (*p* > 0.05).

**Table 4:** **Correlations between nitrite/nitrate levels and clinical periodontal parameters in whole population**.

	**PD**	**PI**	**GBTI**	**GI**	**%GO**	**GCF volume**	**Salivary nitrite**	**Salivary nitrate**	**Plasma nitrite**	**Plasma nitrate**	**GCF nitrite**	**GCF nitrate**
Salivary	*r* = 0.270	*r* = 0.181	*r* = 0.202	*r* = 0.190	*r* = 0.256	*r* = 0.199	–	*r* = −0.010	*r* = −0.002	*r* = −0.063	*r* = 0.000	*r* = −0.120
Nitrite	*p* = 0.006^*^	*p* = 0.067	*p* = 0.040^*^	*p* = 0.053	*p* = 0.009^*^	*p* = 0.043^*^		*p* = 0.923	*p* = 0.987	*p* = 0.527	*p* = 0.996	*p* = 0.226
Salivary	*r* = 0.231	*r* = 0.121	*r* = 0.176	*r* = 0.181	*r* = 0.088	*r* = −0.037	*r* = −0.010	–	*r* = −0.168	*r* = 0.365	*r* = 0.033	*r* = −0.161
Nitrate	*p* = 0.018^*^	*p* = 0.222	*p* = 0.074	*p* = 0.066	*p* = 0.374	*p* = 0.711	*p* = 0.923		*p* = 0.089	*p* = 0.001^**^	*p* = 0.740	*p* = 0.101
Plasma	*r* = 0.081	*r* = 0.023	*r* = 0.027	*r* = 0.053	*r* = 0.060	*r* = 0.030	*r* = −0.002	*r* = −0.168	–	*r* = −0.133	*r* = −0.085	*r* = −0.012
Nitrite	*p* = 0.414	*p* = 0.818	*p* = 0.787	*p* = 0.591	*p* = 0.548	*p* = 0.763	*p* = 0.987	*p* = 0.089		*p* = 0.178	*p* = 0.392	*p* = 0.901
Plasma	*r* = 0.113	*r* = 0.022	*r* = −0.008	*r* = 0.029	*r* = −0.060	*r* = 0.071	*r* = −0.063	*r* = 0.365	*r* = −0.133	–	*r* = −0.025	*r* = −0.054
Nitrate	*p* = 0.253	*p* = 0.826	*p* = 0.933	*p* = 0.772	*p* = 0.543	*p* = 0.474	*p* = 0.527	*p* = 0.001^**^	*p* = 0.178		*p* = 0.802	*p* = 0.589
GCF	*r* = 0.163	*r* = 0.158	*r* = 0.148	*r* = 0.158	*r* = 0.003	*r* = −0.097	*r* = 0.000	*r* = 0.033	*r* = −0.085	*r* = −0.025	–	*r* = −0.267
Nitrite	*p* = 0.099	*p* = 0.109	*p* = 0.134	*p* = 0.109	*p* = 0.973	*p* = 0.327	*p* = 0.996	*p* = 0.740	*p* = 0.392	*p* = 0.802		*p* = 0.006^*^
GCF	*r* = −0.141	*r* = −0.123	*r* = −0.127	*r* = −0.096	*r* = −0.025	*r* = 0.084	*r* = −0.120	*r* = −0.161	*r* = −0.012	*r* = −0.054	*r* = −0.267	–
Nitrate	*p* = 0.154	*p* = 0.213	*p* = 0.199	*p* = 0.332	*p* = 0.803	*p* = 0.398	*p* = 0.226	*p* = 0.101	*p* = 0.901	*p* = 0.589	*p* = 0.006^*^	

*PD, probing depth; PI, plaque index; GI, gingival index; GBTI, gingival bleeding time index; %GO, percentage of gingival overgrowth; GCF, gingival crevicular fluid*.

* *p < 0.05*;

** *p < 0.005*.

## Discussion

To examine the role of NO in the pathogenesis of drug-induced gingival overgrowth and also to determine whether NO levels in plasma, saliva, and GCF can serve as a potential biomarker for the evaluation of drug-induced gingival overgrowth risk, nitrite and nitrate levels in saliva, GCF, and plasma were evaluated in patients receiving either CsA, phenytoin, nifedipine, or diltiazem therapy. To the best of our knowledge, this is the first study that has evaluated the association of plasma, saliva, and GCF nitrite/nitrate levels with drug-induced gingival overgrowth in patients receiving either CsA, phenytoin, nifedipine, or diltiazem therapy.

Although the pathogenesis of drug-induced gingival overgrowth is still uncertain, it is characterized by excessive deposition of extracellular matrix reflecting both elevated synthesis and reduced degradation of matrix components (Seymour et al., 1996). Fibroblasts are well-known to play a critical role in collagen synthesis and connective tissue turnover. The expression of iNOS has been demonstrated in human dermal fibroblasts (Wang et al., 1996). And, it has been reported that iNOS expression and NO production were elevated in keloid scar tissue which is characterized by excess collagen deposition (Hsu et al., 2006). Furthermore, exposure of fibroblasts to exogenous NO resulted in increased expression of collagen in a dose dependent manner (Hsu et al., 2006). Similar to these findings, increased iNOS enzyme activities were detected in overgrown gingiva from patients taking CsA (Gürkan et al., 2009). Additionally, iNOS is largely expressed for sustained periods as a consequence of induction by bacterial lipopolysaccharide and pro-inflammatory mediators such as interleukin 1, tumor necrosis factor-α, and interferon-γ, which are present in inflamed periodontal tissues (Kendall et al., 2001). In drug-induced gingival overgrowth patients due to the abundant gingiva, plaque elimination becomes harder. When plaque accumulates and could not be eliminated, gingival inflammation takes place. Thus, prolonged production of NO by inflammatory cells in gingiva may result in excess collagen deposition via fibroblasts.

In the present study we did not collect whole saliva samples from participants due to the fact that nitrite and nitrate concentrations in whole saliva originate from sources other than the glandular cells, such as GCF and intraoral bacteria. Salivary nitrate is converted to salivary nitrite by oral bacteria (Kleinbongard et al., 2003). Thus, we used pure stimulated parotid saliva for analyzing saliva nitrite and nitrate levels.

In the present study, no significant differences were detected according to age between responders and non-responders in each drug groups. Although age has been suggested as a predisposing factor associated with drug-induced gingival overgrowth (Hefti et al., 1994; Somacarrera et al., 1994), in the present study no significant differences were detected according to age among the study groups for all the drug groups investigated. Similar to our results, Ellis et al. (1999) have reported no relations between age and drug-induced gingival overgrowth.

There were no significant differences in gender ratios between responders and non-responders in all drug groups, except for nifedipine group. In nifedipine group, males were found to be more dominant in patients developing gingival overgrowth, as previously shown before (Hattori et al., 1995; Tavassoli et al., 1998). This difference can be explained with the alteration in androgen metabolism in males.

All periodontal clinical parameters including PI, GI, GBTI, and PD were significantly higher in responders in all drug groups except for diltiazem group. These differences in periodontal clinical parameters between responders and non-responders can be explained by poor plaque control in responders due to the gingival overgrowth. In diltiazem group, although all periodontal clinical parameters were higher in responders, only the GI, GBTI, and %GO scores were significantly different. The reason for the lack of significant differences in PD and PI scores may be the limited number of responders in this drug group.

A total of 104 patients were analyzed separately in four groups according to drugs that patients were taking due to the fact that these drugs may affect directly or indirectly the NO metabolism. Patients receiving nifedipine or diltiazem were not evaluated within the same group although both of the drugs have been known as calcium channel blockers. Because it has been reported that nifedipine had an inhibitory effect on induction of NO synthesis, and also inhibited nitrite production in vascular smooth muscle cells, mesangial cells, and cardiac myocytes. However, diltiazem had no effect on nitrite formation in these three cell types (Hattori et al., 1995). Like diltiazem, it has not been demonstrated such an inhibitory effect of phenytoin on NO production (Nagatomo et al., 2000). On the other hand, it was suggested that CsA could increase gingival iNOS expression (Gau et al., 2005).

The results of the present study demonstrated a significant difference in salivary nitrite and nitrate levels between responders and non-responders in phenytoin group. However, nitrite and nitrate levels in saliva did not differ significantly between responders and non-responders in other drug groups. Similarly, nitrite and nitrate levels in GCF and plasma were not significantly different between subgroups in all study groups.

Although, there have been many studies evaluating the nitrite and nitrate levels of biological fluids as a diagnostic biomarker in oral diseases and periodontitis, only few of them have focused on NO contribution to drug-induced gingival overgrowth. Fu et al. (2000) reported that significantly decreased dimensions of gingival tissues from CsA fed rats receiving L-arginine (NO substrate) or L-NAME (NO blocker) compared to control rats. They also found that plasma nitrite/nitrate concentrations were higher in L-arginine supplement. In another study conducted on rats, it was reported that significantly greater iNOS enzyme activities in gingival tissues obtained from CsA-treated rats than from control rats (Gau et al., 2005). Similarly, the plasma nitrite/nitrate levels were higher in CsA-treated rats than those in control group. In these two studies conducted on rats, nitrite and nitrate were reduced to NO and depicted as total nitrite and nitrate concentration, however nitrite and nitrate concentrations were not reported separately in studied groups. In contrast to the results of these studies, we did not found any significant differences in plasma nitrite and nitrate concentrations between responders and non-responders in all drug groups. In a clinical study, iNOS production significantly increased in connective tissue from gingivitis and CsA-induced gingival overgrowth groups (Gürkan et al., 2009). However, no intergroup differences were found regarding nitrite/nitrate levels in GCF. It was suggested that the reason of this situation might be related to the higher dilution of nitrite and nitrate by GCF volume, which was clearly elevated in responders than non-responders. Another reason might be environmental differences in oral cavity such as composition of oral flora. In the present study, similar to these results, we did not observe any significant difference in nitrite and nitrate levels of GCF between responders and non-responders in all groups.

In the present study, both nitrite and nitrate levels in saliva were found to be significantly increased in responders in phenytoin group. However, no significant differences in salivary nitrite and nitrate levels were observed between responders and non-responders in other drug groups. To the results of a clinical study comparing the NO levels in saliva with the severity of chronic periodontitis, NO levels in saliva increased with severity of chronic periodontitis (Reher et al., 2007). On contrast to these results, Topcu et al. (2014) suggested that nitrite in GCF is a better periodontal disease marker than nitrate in GCF and those in saliva. In another study of the same research group, a tendency to increase in nitrite levels in GCF and peri-implant sulcular fluid with the presence of gingival/peri-implant inflammation was reported (Tözüm et al., 2007).

When the correlations between nitrite and nitrate levels in GCF, saliva, and plasma, periodontal clinical parameters, GCF volume and severity of GO are taken into account, the nitrite level in saliva was found to be significantly correlated to PD and GBTI scores, severity of GO and GCF volume. However, nitrate levels in saliva significantly correlated only to PD score and plasma nitrate level. On the contrary, no significant correlation was found between periodontal clinical parameters, severity of GO and nitrite/nitrate levels in plasma and GCF. Based on these results, it can be suggested that nitrite and nitrate levels in saliva, especially nitrite levels, are more powerful biomarkers than those in plasma and GCF for evaluating drug-induced gingival overgrowth risk. Similar to our results, in a clinical study evaluating the diagnostic role of salivary and GCF nitrite, nitrate and nitric oxide to distinguish healthy periodontium from gingivitis and periodontitis, it was reported that detecting NO biomarker and its end metabolites in saliva is of more value to assess the periodontal health comparing to GCF (Poorsattar Bejeh-Mir et al., 2014). We also found a significant positive correlation between nitrate levels in saliva and plasma, as previously shown before (Clodfelter et al., 2015). The positive correlation between salivary nitrate and plasma nitrate might be explained by the nitrate–nitrite–NO cycle (Lundberg et al., 2008). Ingested nitrate enters the plasma and is taken back up from the circulation by the salivary glands (Lundberg and Govoni, 2004; Lundberg et al., 2008, 2009). Thus, nitrate levels in plasma and saliva are interrelated.

Nitrite and nitrate levels in biological fluids such as plasma, GCF, and saliva might be useful for diagnosis and monitoring of many diseases, including periodontal disease. However, nitrite and nitrate levels in these biological fluids do not always demonstrate the activity and expression of NOS in tissues. It is well-known that NO is produced from L-arginine by NOS in cells (Jenkins et al., 1995). On the other hand, cigarette smoking, oral medicines and supplements that contain nitro-substances, and foods such as spinach, beets, and other green leafy vegetables are exogenous sources of systemic NO (Jobgen et al., 2007). Thus, it is not appropriate to measure nitrite and nitrate levels in biological fluids as an indicator of NO synthesis when these factors are not taken into account.

The most effective treatment of drug-induced gingival overgrowth is substitution of these medications by the patient's physician. The resolution of gingival lesions may take from 1 to 8 weeks depending on the severity of overgrowth (Khocht and Schneider, 1997). However, not all patients respond to this treatment modality, especially those with long-standing gingival overgrowth (Harel-Raviv et al., 1995). Thus, surgical intervention is commonly required to solve the aesthetic and functional problems caused by gingival overgrowth.

## Conclusion

Within the limitations of the present study, nitrite and nitrate levels in saliva could be used as periodontal disease biomarkers in phenytoin-induced gingival overgrowth. And also, saliva has a better diagnostic potential than GCF and plasma for the evaluation of drug-induced gingival overgrowth risk. However, when all drug groups were considered, salivary nitrite and nitrate levels could not be used as a biomarker for drug-induced gingival overgrowth. Additionally, present interpretations require caution due to the limited number of subjects evaluated in drug groups. Further studies including higher subjects will be needed to clarify the use of salivary nitrite/nitrate levels as a biomarker for drug-induced gingival overgrowth.

### Conflict of interest statement

The authors declare that the research was conducted in the absence of any commercial or financial relationships that could be construed as a potential conflict of interest.
